# Comprehensive analysis of mRNAs and miRNAs in the ovarian follicles of uniparous and multiple goats at estrus phase

**DOI:** 10.1186/s12864-020-6671-4

**Published:** 2020-03-30

**Authors:** Xian Zou, Tingting Lu, Zhifeng Zhao, Guangbin Liu, Zhiquan Lian, Yongqing Guo, Baoli Sun, Dewu Liu, Yaokun Li

**Affiliations:** 1 College of Animal Science, South China Agricultural University, Wushan Rd., Tianhe Dist, Guangzhou, 510642 Guangdong Province China; 2 State Key Laboratory of Livestock and Poultry Breeding, Guangdong Key Laboratory of Animal Breeding and Nutrition, Guangdong Public Laboratory of Animal Breeding and Nutrition, Institute of Animal Science, Guangdong Academy of Agricultural Sciences, Guangzhou, 510640 China

**Keywords:** Goat, Follicular development, Kidding rate, RNA-seq

## Abstract

**Background:**

Fertility is an important economic trait in the production of meat goat, and follicular development plays an important role in fertility. Although many mRNAs and microRNAs (miRNAs) have been found to play critical roles in ovarian biological processes, the interaction between mRNAs and miRNAs in follicular development is not yet completely understood. In addition, less attention has been given to the study of single follicle (dominant or atretic follicle) in goats. This study aimed to identify mRNAs, miRNAs, and signaling pathways as well as their interaction networks in the ovarian follicles (large follicles and small follicles) of uniparous and multiple Chuanzhong black goats at estrus phase using RNA-sequencing (RNA-seq) technique.

**Results:**

The results showed that there was a significant difference in the number of large follicles between uniparous and multiple goats (*P* < 0.05), but no difference in the number of small follicles was observed (*P* > 0.05). For the small follicles of uniparous and multiple goats at estrus phase, 289 differentially expressed mRNAs (DEmRNAs) and 16 DEmiRNAs were identified; and for the large follicles, 195 DEmRNAs and 7 DEmiRNAs were identified. The functional enrichment analysis showed that DE genes in small follicles were significantly enriched in ovarian steroidogenesis and steroid hormone biosynthesis, while in large follicles were significantly enriched in ABC transporters and steroid hormone biosynthesis. The results of quantitative real-time polymerase chain reaction were consistent with those of RNA-seq. Analysis of the mRNA-miRNA interaction network suggested that *CD36* (miR-122, miR-200a, miR-141), *TNFAIP6* (miR-141, miR-200a, miR-182), *CYP11A1* (miR-122), *SERPINA5* (miR-1, miR-206, miR-133a-3p, miR-133b), and *PTGFR* (miR-182, miR-122) might be related to fertility, but requires further research on follicular somatic cells.

**Conclusions:**

This study was used for the first time to reveal the DEmRNAs and DEmiRNAs as well as their interaction in the follicles of uniparous and multiple goats at estrus phase using RNA-seq technology. Our findings provide new clues to uncover the molecular mechanisms and signaling networks of goat reproduction that could be potentially used to increase ovulation rate and kidding rate in goat.

## Background

Ovulation rate is a key factor affecting the kidding rate, which is one of the most important economic traits for goat production [1–3]. However, the genetic mechanism of kidding rate associated with ovulation rate is poorly understood, which largely limits the improvement of kidding rate through genetic selection. The major function of the ovary is to produce oocytes for fertilization and secrete steroid hormones for regulating follicular development during the estrus cycle in goat [4]. Oocytes develop and mature in the ovarian follicle, and acquire their developmental competence in follicle through tight bidirectional communication with follicular somatic cells [5, 6]. The follicular somatic cells include epithelial-like granulosa cells (GC), mesenchymal-like theca cells (TC) and cumulus cells (CC), with each type secrets specific regulation factors [7, 8]. GC and TC face each other across a basement membrane. CC are the differentiated GC, which are tightly connected and metabolically coupled with an oocyte via gap junctions and form the cumulus oocyte complex (COC) [9]. The pre-antral follicle is filled with a fluid that is rich in proteins, steroids, and lipids, coming from the blood and secretory activity of follicular somatic cells [10, 11]. The microenvironment of the oocyte has a crucial impact on the acquisition of oocyte developmental competence and possesses molecular factors that are predictive of oocyte developmental potential. However, only 1% of follicles reach ovulation, more than 99% of follicles undergo atresia in mammals [12–16]. In goat, the number of small follicles [S, diameter (d) < 3 mm] far exceeding the number of mid-follicles (d > 3 mm) is a mechanism to regulate the number of oocytes ovulated and to contribute to the timing of ovulation. Hence, it is important to study the contribution of ovarian follicular compartments (follicular fluid, oocyte, CC, GC, and TC) of small follicles and large follicles to the regulation of ovulation number and the timing of ovulation.

In mammals, studies on follicles have mainly been conducted in mice [17], human [18], pig [19], bovine [20], rat [21], and sheep [22]. These studies revealed the effects of GCs and theca cells on follicular development, follicular atresia, and luteal development, and further demonstrated the mechanism of genes and signaling pathways. However, little is known about goat follicles. In goat, previous studies have identified the key genes involved in the regulation of ovulation rate and kidding rate by transcriptome analysis of goat ovaries, as well as the signaling pathways that affect ovulation and fertility [1, 4, 23–29]. Studies on litter size of goats have shown that *PDGFRB*, *MARCH1*, *KDM6A*, *CSN1S1*, *SIRT3*, *KITLG*, *GHR*, *ATBF1*, *INHA*, *GNRH1*, and *GDF9* might be candidate genes for goat reproductive traits [26, 30–39]. Growth hormones and members of the insulin-like growth factor (*IGF*) system (*IGF-I* and *IGF-II*) may play a key role in follicular development and atresia [2, 40], and the genes *FER1 L4* and *SRD5A2* may be associated with the high fecundity of goats [41]. In addition, many studies have suggested that microRNAs (miRNAs) influence ovarian biological processes in goat, and several differentially expressed miRNAs (DEmiRNAs), such as miR-21, miR-99a, miRNA-143, let-7f, miR-493, and miR-200b have been identified and comparatively analyzed in the ovaries of prolific and non-prolifc goats [1, 29, 42]. However, the major genes and miRNAs related to ovulation rate and litter size have not yet been identified in goats through transcriptome sequencing of the ovary as a whole. Hence, since the follicle is a unique microenvironment within which the oocyte can develop and mature into a fertilizable gamete, it is important to individually study single follicles to explore factors that affect ovulation rate and kidding rate in goats.

Chuanzhong (CZ) black goat is an excellent local goat resource in China. The resources are abundant in China as well as Southeast Asia, and play an important role in herbivorous livestock [43]. After long-term natural selection and artificial cultivation, CZ black goat has gradually formed local meat goat breeds with high genetic stability [44]. However, low fecundity remains a key bottleneck limiting the development of goat industry.

To better understand the role and importance of follicles in kidding rate, we performed transcriptome profiling of small follicles (S, d < 3 mm) and large follicles (L, d > 10 mm) from uniparous and multiple CZ black goats at the estrus phase to identify DEmRNAs and DEmiRNAs, respectively. Furthermore, the interaction networks of DEmRNAs and DEmiRNAs were constructed, and Gene Ontology (GO) and Kyoto Encyclopedia of Genes and Genomes (KEGG) enrichment analyses were carried out for DEmRNAs and target genes of DEmiRNAs. In addition, we explored the role of ovarian follicular mRNAs and miRNAs in goat reproduction. Collectively, our findings provide a theoretical basis for improving ovulation and kidding rates in the future.

## Results

### Comparison of follicles between uniparous and multiple CZ black goats

**Fig. 1:**
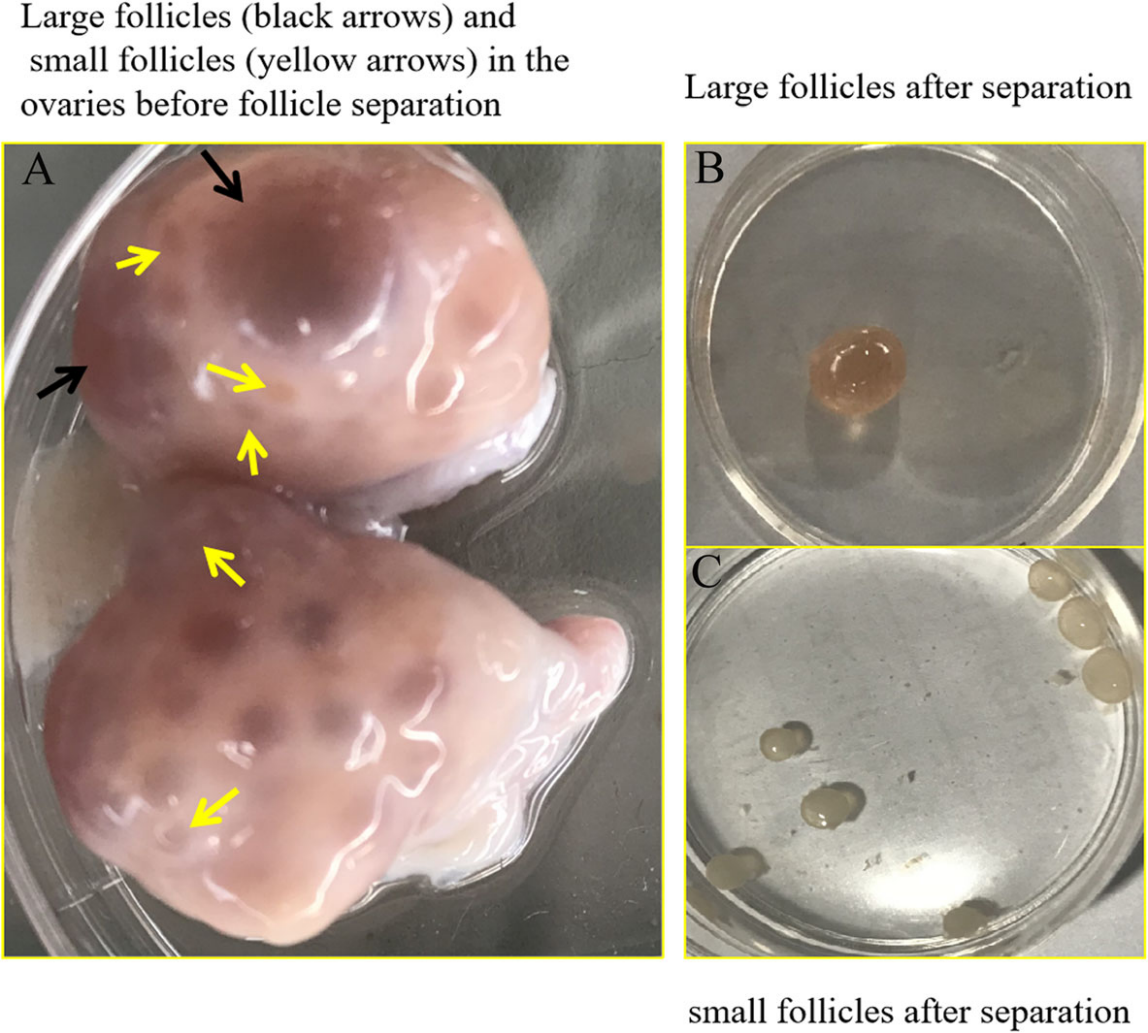
Images of ovaries and follicles. **a** Large follicles (black arrows) and small follicles (yellow arrows) in the ovaries before follicle separation. **b** Large follicles after separation. **c** Small follicles after separation

The follicles around the large follicles were sacrificed during follicle separation, including small follicles (d < 3 mm) and mid-follicles (3 < d < 10 mm). Sometimes the nearby large follicle (d > 10 mm) had to be sacrificed, too. Unfortunately, some small or large follicles were broken up during follicle separation. Finally, eight to fifteen small follicles were isolated from each goat, one to two large follicles were isolated from each uniparous goat, and one to three large follicles each multiple goat. Among the separated follicles used for sequencing, the large follicles (d > 10 mm) were larger in size than the small follicles (d < 3 mm) (Fig. 1a). After separation of follicles, the number of follicles in uniparous and multiple goats was counted and analyzed (Table 1). Multiple goats showed a greater number of follicles than uniparous goats (*P* < 0.05). Furthermore, there was a significant difference in the number of large follicles between uniparous and multiple goats (*P* < 0.05), but no difference in the number of small follicles was observed (*P* > 0.05).

**Table 1:** Comparison of follicles between uniparous and multiple CZ black goats

Groups	The number of follicles		
	Small follicles (d < 3 mm)	Large follicles (d > 10 mm)	Total number^**1**^
Uniparous	30.29 ± 4.36 ^a^	2.71 ± 0.36 ^b^	39.57 ± 3.41 ^b^
Multiple	45.83 ± 7.01 ^a^	4.83 ± 0.7 ^a^	69.17 ± 7.13 ^a^

^1^ The total number of follicles were close to the sum of small (d < 3 mm), mid- (3 < d < 10 mm) and large follicles (d > 10 mm)

Values are expressed as the means ± standard error.

Means within a row with no common superscript letter differ significantly (*P* < 0.05).

### Transcriptome sequencing analysis and mapping

We collected twelve large follicles from four uniparous and four multiple goats, and nine small follicular pools from four uniparous and five multiple goats. The large follicle with few or no blood vessels on the surface were not used to RNA-seq because they considered to be atretic follicles [45, 46]. Eight to ten small follicles were pooled from each goat for a replicate, and only one single large follicle with clear follicular fluid and abundant blood vessels on the surface from each goat for a replicate (Fig. 1b, c). Next, we sequenced the RNA libraries of these seventeen follicular samples (eight large follicles and nine small follicular pools). Clean read counts, mapped ration, and Q30 of sequencing data are displayed in Table 2. The data showed that the sequencing results met the requirements and could be used for further analysis. The expression of 21,343 mRNAs and 436 miRNAs was detected by RNA-sequencing (RNA-seq). Finally, samples were divided into four groups for further analysis: uniparous-small follicles vs multiple-small follicles (Uni-S vs Mul-S) and uniparous-large follicles vs multiple-large follicles (Uni-L vs Mul-L). Unsupervised hierarchical clustering of mRNAs and miRNAs showed that each group clustered together, despite inter-individual variation (Figure S1).

**Table 2:** Q30, clean read counts and mapping ratio of sequencing results

sample	mRNA			miRNA (%)		
	Q30 (%)	Clean Reads (%)	Mapped Ration(%)	Q30 (%)	Clean Reads (%)	Mapped Ration (%)
1 L	94.23	104,768,836 (99.62)	88.85	96.66	23,562,383 (92.40)	74.06
1S	94.26	103,870,160 (99.62)	86.00	96.48	23,729,111 (88.44)	49.21
2 L	94.85	100,396,168 (99.70)	89.57	93.56	18,638,711 (73.47)	40.21
2S	94.88	102,014,414 (99.69)	84.48	93.04	12,918,467 (36.29)	47.62
3 L	94.15	104,879,676 (99.48)	91.70	92.23	22,367,901 (51.67)	67.31
3S	94.24	102,993,890 (99.45)	90.72	91.14	10,392,354 (31.81)	50.17
4 L	92.66	105,470,926 (99.40)	89.70	92.32	12,456,243 (43.15)	49.26
4S	92.27	103,362,624 (99.45)	86.12	94.29	21,982,767 (82.43)	58.40
5 L	92.57	102,209,432 (99.37)	89.67	94.68	21,272,821 (84.74)	67.20
5S	94.11	106,653,668 (99.77)	88.98	93.69	20,242,442 (60.17)	53.04
6 L	93.86	100,346,902 (99.75)	90.32	95.03	18,734,838 (73.61)	48.23
6S	91.52	105,756,846 (99.19)	83.74	94.48	23,270,301 (84.46)	41.44
7 L	92.26	101,449,700 (99.50)	86.32	94.57	20,398,068 (77.25)	35.00
7S	93.79	103,645,956 (99.58)	88.33	95.17	20,963,044 (83.72)	47.44
8 L	91.91	102,150,920 (99.54)	86.68	95.01	22,371,142 (80.09)	47.07
8S	92.38	106,529,902 (99.45)	83.59	94.96	18,992,453 (75.81)	43.75
9S	91.87	105,002,632 (99.51)	86.16	95.2	20,407,498 (87.38)	53.85

*L* large follicles; *S* small follicles

### Identification of DEmRNAs and DEmiRNAs

**Fig. 2:**
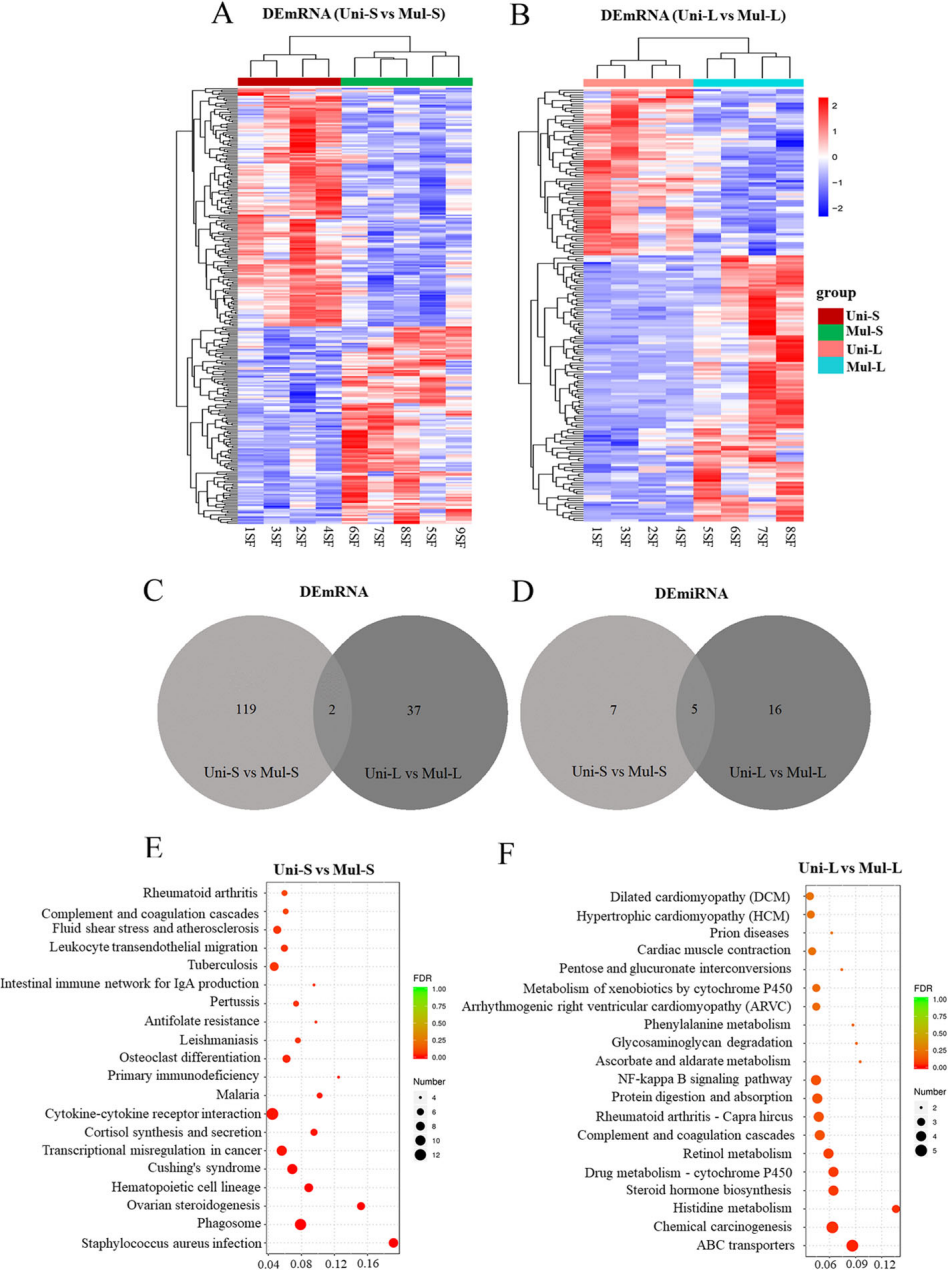
RNA-seq data of DEmRNA expression in large and small follicles from uniparous and multiple goats. **a** Unsupervised clustering analysis showing the expression profiles of DEmRNAs between Uni-S and Mul-S groups. **b** Unsupervised clustering analysis showing the expression profiles of DEmRNAs between Uni-L and Mul-L groups. **c** Venn diagrams demonstrating the distribution of shared DEmRNAs in Uni-S vs Mul-S and Uni-L vs Mul-L groups. **d** Venn diagrams demonstrating the distribution of shared DEmiRNAs in Uni-S vs Mul-S and Uni-L vs Mul-L groups, respectively. **e** Top 20 KEGG pathways of DEmRNAs in Uni-S vs Mul-S. **f** Top 20 KEGG pathways of DEmRNAs in Uni-L vs Mul-L.

DEmRNAs and DEmiRNAs were initially identified by *P* < 0.05 (Additional files 1 and 2). For small follicles, a total of 289 DEmRNAs (131 upregulated and 158 downregulated in multiple goats) and seven DEmiRNAs (seven downregulated in multiple goats) were identified in Uni-S vs Mul-S (Fig. 2a and Table 3a). For large follicles, a total of 195 DEmRNAs (120 upregulated and 75 downregulated in multiple goats) and 16 DEmiRNAs (4 upregulated and 12 downregulated in multiple goats) were identified in Uni-L vs Mul-L (Fig. 2b and Table 3b). For better analysis, the fragments per kilobase million (FPKM) values > 1 of at least three samples per group was used to further quantify the mRNA expression levels. We identified 119 and 37 DEmRNAs from Uni-S vs Mul-S and Uni-L vs Mul-L, respectively, and the top 10 DEmRNAs and DEmiRNAs are shown in Table 3. The venn diagrams of shared DEmRNAs and DEmiRNAs are shown in Fig. 2c, d. Two shared DEmRNAs (*AMDHD1* and *LOC102190765*) and five shared DEmiRNAs (miR-141, miR451-5p, miR-122, miR-182, and miR-206) were identified in both Uni-S vs Mul-S and Uni-L vs Mul-L.

**Table 3:** The top 10 DEmRNAs and DEmiRNAs in Uni-S vs Mul-S (a) and Uni-L vs Mul-L (b)

DEmiRNAs		log2fold change	***P***-value	DEmRNAs	log2fold change	***P***-value
**a**	chi-miR-122	−12.44	1.24E-03	*ARL4C*	−1.59	7.98E-07
	chi-miR-451-5p	−9.01	9.45E-03	*WNT5B*	−1.65	2.25E-06
	chi-miR-206	− 11.91	1.07E-02	*MMP9*	−2.55	3.15E-06
	chi-miR-141	−7.94	1.18E-02	*TGFBI*	−1.55	1.64E-05
	chi-miR-182	−10.62	1.39E-02	*S100A12*	−3.75	5.93E-05
	chi-miR-200a	−7.77	3.18E-02	*INSL3*	2.39	8.34E-05
	chi-miR-184	−11.45	3.83E-02	*MAP7D2*	1.32	8.68E-05
				*LFNG*	−1.35	8.70E-05
				*SDC1*	−1.14	9.74E-05
				*NT5E*	1.03	1.16E-04
**b**	chi-miR-182	−10.83	1.61E-03	*BRINP3*	−1.52	3.59E-04
	chi-miR-122	−10.73	2.17E-03	*DPT*	−1.81	8.07E-04
	chi-miR-133b	−9.62	3.12E-03	*SPOCK2*	− 1.37	1.09E-03
	chi-miR-206	−12.91	5.17E-03	*AMDHD1*	1.19	3.19E-03
	chi-miR-141	−6.46	5.90E-03	*XG*	−1.22	5.27E-03
	chi-miR-34b-5p	−9.82	6.78E-03	*COL6A6*	1.77	6.14E-03
	chi-miR-451-5p	−7.05	6.81E-03	*MFAP5*	−1.67	6.36E-03
	chi-miR-1	−7.02	1.25E-02	*RNASE6*	−1.33	7.43E-03
	chi-miR-496-5p	4.43	2.06E-02	*CCL21*	−1.01	9.18E-03
	chi-miR-34c-5p	−7.86	2.22E-02	*ADAM33*	−1.04	1.01E-02

### Functional annotation of DEmRNAs

DEmRNAs were enriched in biological process, cellular component, and molecular function categories by GO analysis (http://www.geneontology.org/). GO terms with *P* < 0.05 were considered significantly enriched in DEmRNAs. For small follicles, 455 GO terms were significantly enriched, including cell periphery, plasma membrane, steroid biosynthetic process, steroid hydroxylase activity, and receptor binding between the Uni-S and Mul-S groups (Table 4, Additional file 3). For large follicles, 322 GO terms were significantly enriched, including cell periphery, plasma membrane, animal organ development, embryo development, and anion channel activity (Table 4, Additional file 3).

**Table 4:** Top 5 GO terms of DEmRNAs in Uni-S vs Mul-S and Uni-L vs Mul-L

Term	gene count	***P***-value	Term	gene count	***P***-value
**Uni-S vs Mul-S**			**Uni-L vs Mul-L**		
**Biological process**					
Immune system process	45	3.10E-07	Anterior/posterior pattern specification	11	1.20E-07
Immune response	24	6.90E-05	Definitive hemopoiesis	4	9.30E-06
Steroid biosynthetic process	7	1.20E-04	Regionalization	11	1.10E-05
Organic hydroxy Compound biosynthetic process	8	2.40E-04	Pattern specification process	12	1.10E-05
Superoxide anion generation	4	3.00E-04	Skeletal system morphogenesis	9	1.80E-05
**Cellular component**					
Cell surface	18	4.40E-05	Extracellular region part	13	4.60E-04
Extracellular region	24	7.00E-05	Extracellular region	14	5.70E-04
Extracellular space	18	1.20E-04	Extracellular matrix	5	2.38E-03
Extracellular region part	21	1.70E-04	Cell periphery	25	6.60E-03
Plasma membrane	45	1.08E-03	Protein C inhibitor-TMPRSS7 complex	1	6.88E-03
**Molecular function**					
Deaminase activity	3	4.90E-04	Icosanoid receptor activity	2	1.20E-03
Receptor activity	17	6.30E-04	Chloride channel activity	3	2.70E-03
Hydrolase activity, acting on carbon-nitrogen (but not peptide) bonds, in cyclic amidines	3	6.40E-04	Drug binding	3	4.20E-03
Glycogen binding	2	6.50E-04	Chloride transmembrane transporter activity	3	4.20E-03
Receptor binding	24	6.70E-04	Anion channel activity	3	4.80E-03

DEmRNAs were also plotted to KEGG reference pathways (https://www.kegg.jp/kegg/pathway.html). The significantly enriched KEGG pathways (*P* < 0.05) were listed in Additional file 4. Of these KEGG pathways, ovarian steroidogenesis, cortisol synthesis and secretion, cytokine-cytokine receptor interaction, steroid hormone biosynthesis, and metabolism of xenobiotics by cytochrome P450 were significantly enriched between the Uni-S and Mul-S groups (Fig. 2e), and ABC transporters, retinol metabolism, steroid hormone biosynthesis, drug metabolism-cytochrome P450, and metabolism of xenobiotics by cytochrome P450 were significantly enriched between the Uni-L and Mul-L groups (Fig. 2f).

### DEmRNA-DEmiRNA interaction network

**Fig. 3:**
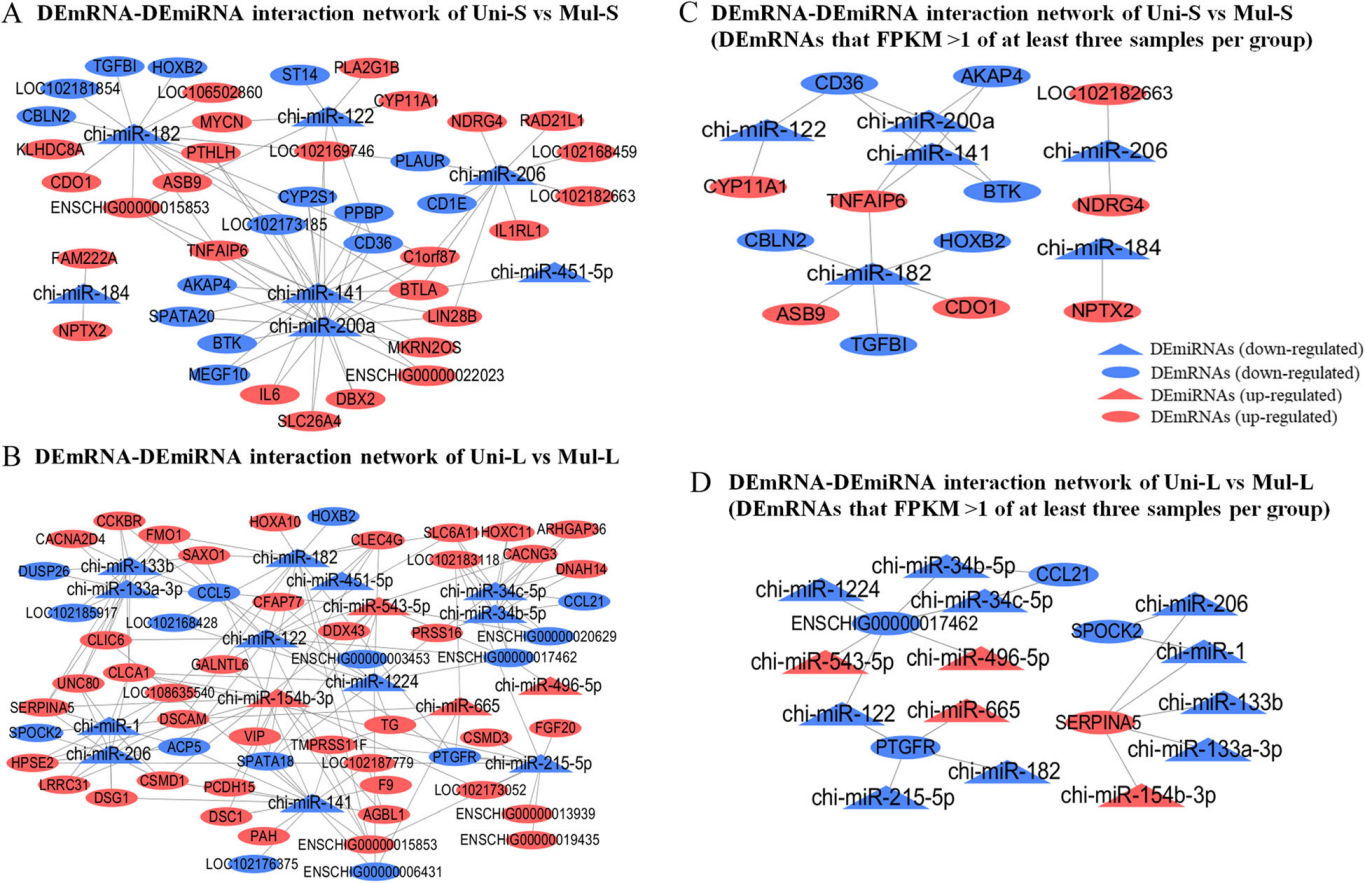
The original and selected DEmRNA-DEmiRNA interaction network of Uni-S vs Mul-S and Uni-L vs Mul-L. **a** The original DEmRNA-DEmiRNA interaction network of Uni-S vs Mul-S. **b** The original DEmRNA-DEmiRNA interaction network of Uni-L vs Mul-L. **c** The selected DEmRNA-DEmiRNA interaction network of Uni-S vs Mul-S. DEmRNAs were screened according to FPKM > 1 of at least three samples per group. **d** The selected DEmRNA-DEmiRNA interaction network of Uni-L vs Mul-L. DEmRNAs were screened according to FPKM > 1 of at least three samples per group. Ellipses and triangle represent DEmRNAs and DEmiRNAs, respectively. Red and blue colored nodes represent upregulation and downregulation, respectively

For small follicles (Uni-S vs Mul-S), the miRNA–mRNA target prediction analyses identified 76 miRNA–mRNA target pairs, including only seven significant miRNAs, chi-miR-200a (degree = 20, degree means the number of DEmiRNAs’target genes), chi-miR-141 (degree = 20), chi-miR-182 (degree = 16), chi-miR-206 (degree = 10), chi-miR-122 (degree = 7), chi-miR-184 (degree = 2) and chi-miR-145-5p (degree = 1) (Fig. 3a). For large follicles (Uni-L vs Mul-L), a total of 153 possible significant miRNA–mRNA interaction pairs were obtained; chi-miR-141 (degree = 17), chi-miR-182 (degree = 13), chi-miR-122 (degree = 12) and chi-miR-154b-3p (degree = 12) were the top DEmiRNAs that had most target genes (Fig. 3b).

In order to further narrow the scope of genes and obtain more meaningful candidate genes, DEmRNAs were screened according to FPKM > 1 of at least three samples per group. Then, 19 pairs and 19 pairs of DEmRNAs-DEmiRNAs were obtained from Uni-S vs Mul-S and Uni-L vs Mul-L, respectively (Fig. 3c, d). Among them, in Uni-S vs Mul-S, *TNFAIP6* (degree = 3) was upregulated in multiple goats; *CD36* (degree = 3), *BTK* (degree = 2) and *AKAP4* (degree = 2) were downregulated in multiple goats. In Uni-L vs Mul-L, *SERPINA5* (degree = 5) was upregulated in multiple goats; *ENSCHIG00000017462* (degree = 6) and *PTGFR* (degree = 4) were downregulated in multiple goats.

### Validation by quantitative real-time polymerase chain reaction (qRT-PCR)

**Fig. 4:**
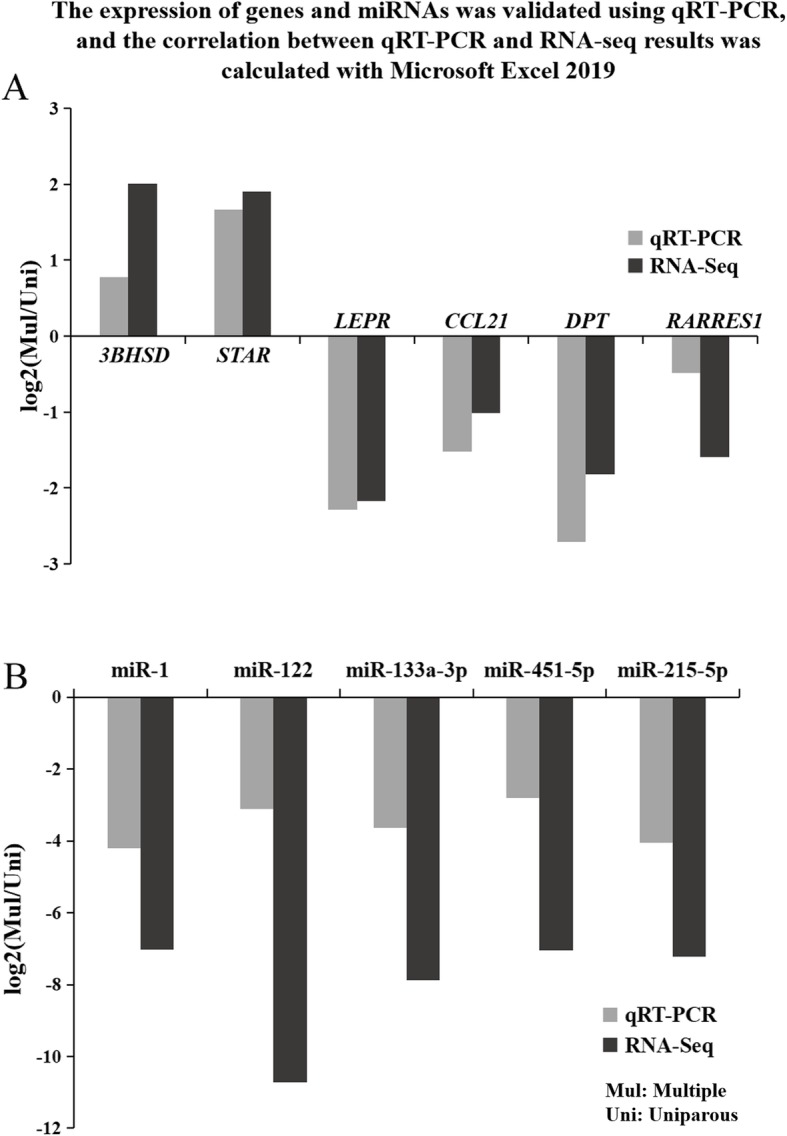
Verification of differently expressed genes and miRNAs by qRT-PCR. **a** The expression level of six genes were validated by qRT-PCR and compared with the results of RNA-seq. *3BHSD*, *STAR* and *LEPR* were selected from Uni-S vs Mul-S, and *CCL21*, *RARRES1* and *DPT* were selected from Uni-L vs Mul-L. **b** The expression level of five miRNAs were validated by qRT-PCR and compared with the results of RNA-seq. These five DEmiRNAs were selected from Uni-L vs Mul-L. Data were presented as expression values of genes and miRNAs in Uni-S vs Mul-S and Uni-L vs Mul-L. For qRT-PCR data, mRNA expression was normalized to *β-actin* in the same cDNA sample, and miRNA expression was normalized to U6

A total of 6 DEmRNAs and 5 DEmiRNAs were selected for verification by qRT-PCR. Based on the RNA-seq results, *3BHSD* and *STAR* expression were upregulated and *LEPR* expression was downregulated in small follicles of multiple goat, and *CCL21*, *RARRES1*, and *DPT* expression were downregulated in large follicles of multiple goat. Notably, genes *3BHSD* and *STAR* are both involved in the four significant pathways (ovarian steroidogenesis, cushing’s syndrome, cortisol synthesis and secretion and aldosterone synthesis and secretion), *LEPR* gene is involved in the two significant pathways (cytokine-cytokine receptor interaction and neuroactive ligand-receptor interaction). Five DEmiRNAs were downregulated in multiple large follicles. Based on the qRT-PCR results, expression of these 6 DEmRNAs and 5 DEmiRNAs were consistent with that in the RNA-seq results (Fig. 4).

## Discussion

Exploring the genetic mechanism associated with ovulation rate is important to improve kidding rate, which is fundamental to goat production. The greater number of large follicles that stimulate ovulation is believed to be the primary reason for higher ovulation rate [39]. Although several mRNAs and miRNAs have been found to play critical roles in ovarian biological processes, the majority of the genes and miRNAs related to ovulation rate and kidding rate have not been identified in goats. In addition, the interaction between mRNAs and miRNAs in follicular development is not yet completely understood. Hence, we compared DEmRNAs and DEmiRNAs from different size follicles between uniparous and multiple CZ black goats at estrus phase using RNA-seq. The results showed that the number of large follicles in multiple goats was significantly higher than that in uniparous goats (*P* < 0.05), while no difference in the number of small follicles was observed between uniparous and multiple goats, verifying that the greater number of large follicles was related to higher ovulation rate [39]. Based on the RNA-seq data, we identified 119 and 37 DEmRNAs by comparing Uni-S with Mul-S and Uni-L with Mul-L, respectively (FPKM> 1 of at least three samples per group). These DEmRNAs were found to be involved in various ovarian development-related pathways, such as ovarian steroidogenesis, steroid hormone biosynthesis, and metabolism of xenobiotics by cytochrome P450, etc. (Fig. 2e, f). Approximately 37% of the DEgenes in the Uni-S vs Mul-S group and 41% in the Uni-L vs Mul-L group were reported to be associated with mammalian reproduction, such as *TNFAIP6*, *MMP9*, *INSL3*, *LEPR*, *3BHSD*, *LHCGR*, *ARL4C*, *CD36*, *CYP11A1*, *AMDHD1*, *SPOCK2*, *AMDHD1*, *MFAP5*, *CCL21*, *PTGFR*, and *SERPINA5* [23, 47–60]. Of these genes, *TNFAIP6, CYP11A1*, *CD36*, *PTGFR*, and *SERPINA5* were found to be associated with the ovulation rate. In addition, *TNFAIP6, CYP11A1* and *CD36* were found to be differentially expressed when comparing Uni-S vs Mul-S, while *PTGFR* and *SERPINA5* were found to be differentially expressed when comparing Uni-L vs Mul-L.

TNFAIP6 is a secretory protein of the hyaluronan-binding protein family that played a role in CC stabilization and expansion, and it was upregulated in bovine GCs during ovulation [47, 61–63]. *TNFAIP6*-deficient female mice were sterile [44]. The present study reported that *TNFAIP6* expression was 8-fold higher in Mul-S than in Uni-S, suggesting a possible role of *TNFAIP6* in CC expansion in the small follicle of multiple goats during the estrus phase. CYP11A1 played a key role in the regulation of steroid-producing pathways in GCs [64]. The first step in steroid biosynthesis was the conversion of cholesterol into pregnenolone through the action of CYP11A1 in the mitochondria, and then pregnenolone acted as a substrate for progesterone synthesis through the mediation of *3b-HSD* expression [65, 66]. In this study, *CYP11A1* expression was found to be upregulated in the small follicle of multiple goats, which was consistent with previous reports [67]; this observation demonstrates that *CYP11A1* might play multiple roles in goat ovarian development. CD36 was a multifunctional receptor-binding autocrine growth factor that could regulate angiogenesis, cell growth, and adhesion. The expression of *CD36* was found to be follicle-type dependent with the greatest expression in atretic follicles, and the lowest expresssion in healthy follicles [68–71]. *CD36* knockdown has been shown to increase proliferation and expression of survival and angiogenic markers in GCs [70]. In this study, *CD36* expression was shown to be downregulated in multiple goats and it was associated with specific hematopoietic cell lineages, suggesting that the low expression of *CD36* expression in multiple individuals may promote follicular maturation by stimulating GCs proliferation or angiogenesis, which then increases the kidding rate. Overall, *TNFAIP6* and *CYP11A1* played a positive role in the regulation of ovulation, while *CD36* contributed to follicular atresia. Accordingly, *TNFAIP6* and *CYP11A1* expression was upregulated in the small follicles of multiple goats, while *CD36* expression was downregulated, indicating that there were more small follicles that could grow into dominant follicles in multiple goats. Thus, these genes might play a key role in the ovulation rate or kidding rate in goats.

PTGFR was a regulatory factor in follicular development that affected mammalian reproductive pathways [72–74]. In the GCs of periovulatory follicles of mice, the expression of *PTGFR* was shown to be drastically reduced [75].. Our results showed that *PTGFR* expression was downregulated in the large follicle from multiple goats, suggesting that the greater reduction of *PTGFR* expression observed in multiple goats may contribute to a the higher ovulation rate. Previous studies have reported that SERPINA5, a protease inhibitor, was expressed in the reproductive tract of adult mice and in the GCs of bovine follicles and that was highly expressed in bovine healthy follicles [60, 76]. *SERPINA5* expression was shown to be downregulated in ovarian cancer (OC) [77–79]. Here, we found that *SERPINA5* expression was upregulated in the large follicles of multiple goats, suggesting that *SERPINA5* may affect follicular development and the kidding rate in goats. Taken together, downregulation of *PTGFR* and upregulation of *SERPINA5* in large follicles may represent useful strategies for increasing the ovulation rate in multiple goats.

We identified 17 and 16 DEmiRNAs in the Uni-S vs Mul-S and Uni-L vs Mul-L groups, respectively. The miRNAs, miR-200a, miR-451-5p, miR-141, miR-182, miR-206, and miR-122 were highly expressed in the small follicles of multiple goats, while miR-1, miR-206, miR-133a-3p, miR-133b, miR-182, miR-215-5p, miR-122, and miR-451-5p were highly expressed in the large follicles of multiple goats. However, only miR-200a has been previously reported to be highly expressed in goat ovaries [1], demonstrating that the the independence of the expression of miRNAs in whole ovary, small follicles, and large follicles. Of these 14 highly expressed DEmiRNAs, miR-200a, miR-141, miR-1, miR-206, miR-133b, miR-133a-3p, miR-182, and miR-122 have been reported to play important roles in basic reproductive activities. miR-200a was frequently overexpressed and is closely related to the migratory, proliferative, and invasive abilities of OC cells [80–82]. miR-141 was shown to be significantly upregulated in OC cell lines and advanced metastatic OC [83, 84], and can inhibit GC apoptosis by targeting *DAPK1* through the MAPK signaling pathway, leading to the development of polycystic ovary syndrome (PCOS) [85]. In this study, miR-200a and miR-141 expression were upregulated in the small follicles of uniparous goats, suggesting that these miRNAs might affect normal GC development, thereby affecting follicular development. miR-206 and miR-1 were potential tumor suppressors that have been shown to be downregulated in OC tissues, to inhibit *c-Met* expression, and to regulate cell proliferation, migration, and invasion [86, 87]. And miR-206 has also been shown to induce apoptosis [88, 89]. Here, miR-206 and miR-1 expression was downregulated in the large follicles of multiple goats, which may be related to oocyte maturation and ovulation in goats. Members of the miR-133 family (miR-133a-3p and miR-133b) have been shown to be involved in the regulation of various cellular processes, such as cell proliferation, apoptosis, migration, and invasion [90, 91]. *FOXL2* was a conserved, early-acting gene in vertebrate ovarian development, and it played an important role in GC proliferation and oocyte maturation [68, 69]. Recently, *FOXL2* expression has been reported to be regulated by miR-133b. miR-133b can bind to the *FOXL2–3′UTR* in GCs to inhibit the expression of the downstream genes, *STAR* and *CYP19A1*, which simultaneously promoted estrogen secretion in GCs [92]. miR-133b has been shown to be upregulated more than 30-fold in metaphase I oocytes after IGF-1 treatment, and it may play important roles in oocyte growth and maturation by regulating the expression of its potential target gene *TAGLN2* [93].

Comparing Uni-S with Mul-S and Uni-L with Mul-L, miR-182 and miR-122 were found to be differentially expressed in both groups. miR-182 was shown to be upregulated in the follicular fluid of patients with PCOS [94], and its expression was significantly increased in OC cell lines [95]. miR-122 inhibited epithelial mesenchymal transition by regulating *P4HA1* expression in OC cells [43]. In addition, miR-122 regulated *LHCGR* expression, which was crucial for mediating LH action in growing follicles via modulating LRBP levels during FSH-induced follicle growth [96]. miR-122 has been shown to increase *LHR* mRNA levels by modulating the expression of *LRBP* through the regulation of *SREBP* activation, which was crucial for regulating key reproductive processes, such as ovulation and CL function [97]. In this study, both miR-182 and miR-122 were upregulated in uniparous goats, suggesting that they may affect the ovulation rate by affecting follicular growth in uniparous goats.

Taken together, the genes *CD36*, *TNFAIP6*, *CYP11A1*, *SERPINA5*, and *PTGFR* were reported to be crucial for regulating the proliferation, migration, invasion and apoptosis of follicular somatic cells. The present study showed that *TNFAIP6, CYP11A1*, and *CD36* were found to be differentially expressed when comparing Uni-S with Mul-S, and *PTGFR* and *SERPINA5* were found to be differentially expressed when comparing Uni-L with Mul-L, suggesting that they may affect follicular development by affecting the growth of follicular somatic cells in goats. Furthermore, we predicted the target genes for DEmiRNAs and some of the targets showed high expression including *CD36* (miR-122, miR-200a), *TNFAIP6* (miR-200a, miR-182), *CYP11A1* (miR-122), *SERPINA5* (miR-1, miR-206, miR-133a-3p, and miR-133b), and *PTGFR* (miR-182 and miR-122); these expression regulation mechanisms may be related to ovulation and kidding rates.

## Conclusions

Identifying the specific subset of genes and miRNAs involved in follicular development is essential for fully comprehending the cascade of events leading to ovulation and will likely contribute to an improved ability to control fertility. This study was the first to reveal the DEmRNAs and DEmiRNAs as well as their interaction in the follicles of uniparous and multiple goats at the estrus phase using RNA-seq technology. Numerous DEmiRNAs were more highly expressed in the small follicular libraries of multiple goats (miR-200a, miR-451-5p, miR-141, miR-182, miR-206, and miR-122), and while other DEmiRNAs were more highly expressed in the large follicular libraries of multiple goats (miR-1, miR-206, miR-133a-3p, miR-133b, miR-182, miR-215-5p, miR-122 and miR-451-5p). The higher expression of *TNFAIP6*, *CYP11A1* and *CD36* in the small follicles of multiple goats, and the higher expression of *PTGFR* and *SERPINA5* in the large follicles of multiple goats may play a critical role in goat prolificacy. Our findings provide a basic foundation for elucidating the regulatory mechanisms of mRNAs and miRNAs in CZ black goats and a unique source for exploring the corresponding targets of the miRNAs in the future.

## Methods

### Animals and sample preparation

In the present study, CZ goats were obtained from the South China Agriculture University, Guangdong, China. A total of eleven healthy female goats of the same age (about 3.5–4.5 years old) with more than three litters were raised under natural light conditions with free access to food and water. Five goats were uniparous with only one kid per birth, and six goats were multiple with an average of 2.8 kids per birth. Mul: the six goats had three litters whose kidding ≥2. Uni: the five goats had three litters whose kidding = 1.

In order to achieve estrus synchronization, each goat was injected intramuscularly with 0.1 mg chloroprostenol. Eighteen days later, male goats (vasectomy and ligation of vas deferens) were used to confirm whether the estrous was synchronized. At 24 h after estrus (in the middle of estrus), all goats were weighed and slaughtered at a local slaughterhouse. The intact ovaries were rapidly collected and washed with 75% alcohol thrice. Then they were soaked into phosphate buffered saline (PBS). The follicles were achieved by using micro-blades and tweezers under surgical dissecting microscope within 30 min. Isolated follicles from each ovarian were washed with PBS to eliminate debris, then froze in liquid nitrogen instantly and stored at − 80 °C for generating RNA libraries. The small follicles (S, d < 3 mm) or large antral follicles (L, d > 1 cm) were isolated from the ovarian stromal tissues with tweezers by immersing the ovary in PBS, and then placed in liquid nitrogen (Table 5). The small and large follicles were collected at the same time within 30 min.

**Table 5:** Sample characteristics

Index	Number ^**a**^(n)	Age (years old)	S^**b**^ (d < 3 mm) /per one^**c**^	L^**d**^ (d > 1 cm) /per one^**e**^
Uniparous (Uni)	5	3.5–4.5	8–10	1–2
Multiple (Mul)	6	3.5–4.5	8–10	1–3

^a^ The number of uniparous (Uni) and multiple (Mul) goats selected in this experiment.

^b^ Small follicles

^c^ Eight to ten small follicles were collected and pooled from each goat

^d^ Large follicles

^e^ One to three large follicles were collected from each goat

### RNA extraction and qualification

Total RNA was extracted from the whole ovarian follicle using TRIzol reagent (Invitrogen, Carlsbad, CA, USA) according to the manufacturer’s instructions. RNA quality was evaluated with the NanoDrop ND-2000 spectrophotometer (Thermo Fisher Scientific, Wilmington, DE, USA) and Agilent 2100 Bioanalyzer (Agilent Technologies, Palo Alto, CA, USA). RNA integrity was evaluated using 1% agarose gel. Purified RNA was stored at − 80 °C until further use. RNA with amount > 6 μg, concentration ≥ 200 ng/mL, 1.8 < OD260/280 < 2.2, and RNA integrity number (RIN) > 8.5 was used for the preparation of cDNA libraries.

### mRNA sequencing and data processing

A total of 3 μg RNA per sample was used as input material for removing ribosomal RNA using the Ribo-Zero Magnetic kit (EpiCentre, Madison, WI, USA). RNA was then fragmented into 200–300 bp by ion interruption. The first cDNA strand was synthesized using 6-base random hexamer primers and reverse transcriptase, and the second cDNA strand was synthesized with dUTP instead of dTTP. The library was constructed and amplified according to the size of the fragments (300–400 bp) by PCR using the Agilent 2100 Bioanalyzer (Agilent Technologies, Palo Alto, CA, USA). The hybrid library was uniformly diluted to 2 nM through proportionally mixing the libraries containing different index sequences and forming a single chain library using TruseqTM RNA sample prep kit (Illumina, San Diego, CA, USA). The libraries were sequenced by paired-end sequencing on the HiSeq 2500 sequencer (Illumina, San Diego, CA, USA).

By using base calling, raw data obtained from high-throughput RNA-seq were translated into raw FASTQ sequence data. Raw reads of FASTQ format were then processed with Perl scripts to assess the quality of data used for subsequent analysis. To obtain clean reads from RNA-seq data for mRNA analysis, low quality sequences, including adaptor sequences, sequences with quality score < 20, and sequences with N base rate of raw reads > 10% were removed using cutadapt (http://cufflinks.cbcb.umd.edu/). Next, low quality sequences, including adaptor sequences, sequences with quality score < 20, sequences with N base, and sequences less than 18 bp were removed using Fastx-Toolkit (http://hannonlab.cshl.edu/f astx toolkit/). After filtering the raw reads, clean reads were obtained. Statistical analysis was performed to evaluate its quantity and quality, including Q30 (the percentage of the number of bases with phared score > 30 in the original data to the total number of bases) statistics, data quantity statistics, base content statistics, etc.

After trimming the raw reads, the clean reads were mapped to the goat reference genome (GCF_001704415.1_ARS1) Ensembl V96 using Tophat2. The mismatch of default reads and reference genomic sequence was within 2, and the mapping ratio is generally higher than 70%.

### Small RNA library construction, sequencing, and data processing

Following extraction and purification, about 2 μg of total RNA per sample was used to construct the small RNA library using the TruSeq Small RNA Sample Prep Kit (Illumina, San Diego CA, USA). All libraries for high-throughput sequencing of miRNA were amplified using PCR by adding the sequencing connector and the index part. Next, the 18–36 nucleotide RNA was purified using 6% Novex TBE PAGE gel (1.0 mm, 10 well) and quantified using the Agilent 2100 Bioanalyzer. Single-stranded cDNA template was subjected to bridge PCR followed by Illumina single-end sequencing on the HiSeq 2500 sequencer (Illumina, San Diego, CA, USA).

Raw reads were processed with the script developed by consisting of index trimming, read alignment, and read counting. To obtain clean reads, raw reads were further filtered according to the following rules: (1) Removing low quality reads containing more than one low quality (Q-value ≤20) base or containing unknown nucleotides (N); (2) Removing reads without 3′ adapters; (3) Removing reads containing 5′ adapters; (4) Removing reads containing 3′ and 5′ adapters but no small RNA fragment between them; (5) Removing reads containing poly A in the small RNA fragment; and (6) Removing reads shorter than 18 nt (not including adapters). The resected clean reads were mapped to the goat reference genome (GCF_001704415.1_ARS1) Ensembl V96 using miRDeep2, in which the mapper.pl program invokes Bowtie for the alignment between the de-repeat sequence and the reference genome sequence. The de-repeat sequences were aligned to the mature miRNA and precursor miRNA sequences of the species in the miRBase (http://www.mirbase.org/) [98], and the detected miRNA was annotated. Using mireap to analyze unannotated sequences of information, a new miRNA prediction analysis was carried out. According to the number of mature miRNA sequences of this species, the read count values of miRNA were calculated.

### Identification of DEmRNAs and DEmiRNAs

For RNA-seq analysis, the criteria for measuring mRNA and miRNA expression levels were FPKM and CPM (CPM = C/N × 1,000,000; C is the total number of reads mapped onto the gene, and N is the total number of mapped reads) values, respectively. Differential expression in each group was identified using DESeq version 1.18.0 with R package, and DEmRNAs and DEmiRNAs were identified with the cut-off criteria of |log_2_FoldChange| > 1 and *P*-value < 0.05.

The volcano plots of differentially expressed genes were generated using the ggplots2 package in R. All genes and samples were clustered using the heatmap package in R software. The Euclidean distance was calculated based on the expression level of the same gene in different samples and the expression patterns of different genes in the same sample. The complete linkage hierarchical clustering method, which uses the largest intercluster distance, was used for clustering. Shared DEmRNAs or DEmiRNAs were identified in both Uni-S vs Mul-S and Uni-L vs Mul-L groups.

### Prediction of the target genes of the DEmiRNAs and construction of mRNA-miRNA interaction network

Systematic bioinformatic analysis was developed based on possible functional relationships between DEmiRNAs and DEmRNA. In this study, miRanDa was used to predict the target genes of DEmiRNAs using the 3’UTR mRNA sequence of the species as the target sequence. The parameters used to determine miRNA-target predicted interaction were the mapping score more than 140 and the free energy less than 1.0. Due to multiple target genes, data were re-filtered and the criterion was that the gene could only be retained in FPKM > 1 of at least three samples per group. Based on the re-filtered data, DEmRNA-DEmiRNA pairs in Uni-S vs Mul-S and Uni-L vs Mul-L were constructed and visualized using Cytoscape (v3.5.1) software.

### Functional enrichment analysis

GO (http://geneontology.org/) and KEGG (http://www.kegg.jp/) pathway enrichment analysis were used to analyze DEmRNAs and target genes of DEmiRNAs. GO and KEGG pathway analysis of the differentially expressed and target genes was performed with the software DAVID (https://david.ncifcrf.gov). Degree of enrichment was measured by Rich factor, FDR, and the number of genes that were enriched in the pathway. Both GO terms and KEGG pathways were corrected. A *P*-value ≤0.05 was considered to be significantly enriched.

### RNA preparation and qRT-PCR

First, total RNA was extracted from the ovarian follicles with the Total RNA Kit II (OMEGA, USA) for qRT-PCR analysis of DEmRNAs and DEmiRNAs. Second, the levels of DEmRNAs were measured using the PrimeScript® RT Reagent Kit with gDNA Eraser (TaKaRa, China) and qRT-PCR was performed using SYBR® Green PCR Supermix (Bio-Rad, USA). Each 20 μL reaction included 10 μL of SYBR® Green PCR Supermix, 1 μL of each divergent primer, 1 μL of cDNA, and 7 μL of RNase-free water. The cycling conditions included an initial single cycle (95 °C for 1 min), followed by 34 cycles of 95 °C for 30 s, 58 °C for 30 s, and 72 °C for 1 min. Melting-curve analysis was performed to verify the product identity. The levels of DEmiRNAs were then measured by qRT-PCR using miDETECT A Track™ miRNA qRT-PCR Starter kit (RiboBio, Guangzhou, China). Each 20 μL reaction included 10 μL of SYBR Green Mix, 0.5 μL of each divergent primer, 1 μL of cDNA, and 8 μL of RNase-free water. The cycling conditions included an initial single cycle (95 °C for 10 min), followed by 40 cycles of 95 °C for 5 s, 60 °C for 30 s, and 72 °C for 30 s. Melting-curve analysis was performed to verify the product identity.

According to the results of RNA-Seq, 6 DEmRNAs (FPKM > 1) and 5 DEmiRNAs (CPM > 1) were selected for validation by qRT-PCR. Primers for DEmRNAs were designed by Premier 5 and obtained from Sangon Biotech (Shanghai, China), and primers for DEmiRNAs were designed and obtained from RiboBio Company (Guangzhou, China). Information regarding the quantitative primers used in this experiment is listed in Table 6. β-actin and U6 were used as endogenous controls for mRNA and miRNA, respectively, and all reactions were performed in triplicate. Relative expression levels were calculated using the 2^−ΔΔCt^ method. The correlation between qRT-PCR and RNA-seq results was calculated with Microsoft Excel 2019.

**Table 6:** Primers of DEmRNAs

Gene name	Forward primer (5′-3′)	Reverse primer (5′-3′)	Product length/bp	Annealing temperature	mRNA version
*3BHSD*	agggcatctcagtggtca	ggataaagactggcacgcta	144	57.4 **°C**	NM_001285716.1
*LEPR*	ccattgagaagtatcagttcagtc	catgctggtgtttttcatcatcttg	105	58.4 **°C**	XM_018045220.1
*STAR*	cagaagggtgtcatcagagc	tgagcagccaggtgagttt	97	58.6 **°C**	XM_013975437.2
*CCL21*	ccgaaagaagattcccgcca	ggcgagaacaggatagctgg	90	60.1 **°C**	XM_005684096.3
*RARRES1*	gcgcgtgggttaatcagaag	acattaacagctggtctgggtt	148	59.8 **°C**	XM_018048385.1
*DPT*	gtaccagacatgctccaacaa	ctgttgtcagccagcaggaa	137	59.4 **°C**	XM_005690613.3
*β-actin*	tgcttctaggcggactgatt	tacaatcaaagtcctcggccac	106	59.7 **°C**	NM_001314342.1

### Statistical analysis

One-way analysis of variance was conducted using JMP 8.0 software (SAS Institute, Cary, NC). All results were expressed as means ± standard error, and *P*-values below 0.05 were considered to indicate statistically significant differences.

## Supplementary information

**Additional file 1 Figure S1**, RNA-seq reveals distinct expression pattern of mRNAs and miRNAs among the four groups. (A) Unsupervised clustering analysis showing expression profiles of mRNAs between Uni-S and Mul-S groups. (B) Unsupervised clustering analysis showing expression profiles of mRNAs between Uni-L and Mul-L groups. (C) Unsupervised clustering analysis showing expression profiles of miRNAs between Uni-S and Mul-S groups. (D) Unsupervised clustering analysis showing expression profiles of miRNAs between Uni-L and Mul-L groups.

**Additional file 2.** RNA-seq data of DEmRNA expression in large and small follicles from uniparous and multiple goats.

**Additional file 3.** RNA-seq data of DEmiRNA expression in large and small follicles from uniparous and multiple goats.

**Additional file 4.** The significant Go term of DEmRNAs in large and small follicles from uniparous and multiple goats.

**Additional file 5.** The significant KEGG pathways of DEmRNAs in large and small follicles from uniparous and multiple goats.

## Data Availability

We have submitted the sequencing data to NCBI SRA repository under the BioProject ID PRJNA579007 and PRJNA579194.

## Abbreviations

miRNAs: MicroRNAs
CZ black goat: Chuanzhong black goat
FPKM: Fragments Per Kilobase of exon model per Million mapped reads
GO: Gene ontology
KEGG: Kyoto encyclopedia of genes and genomes
RNA-seq: RNA sequencing
qRT-PCR: Quantitative real time polymerase chain reaction
DEmiRNA: Different expressed miRNA
DEmRNA: Different expressed mRNA
GH: Growth hormone
GCs: Granulosa cells
CCs: Cumulus cells
TCs: theca cells
CL: Corpus luteum
OC: Ovarian cancer
PCOS: Polycystic ovary syndrome
