# Supplementary figures and images for: Comprehensive analysis of mRNAs and miRNAs in the ovarian follicles of uniparous and multiple goats at estrus phase
Source: BMC Genomics. 2020 Mar 30;21:267. doi: 10.1186/s12864-020-6671-4 (PMC7106838; doi:10.1186/s12864-020-6671-4)

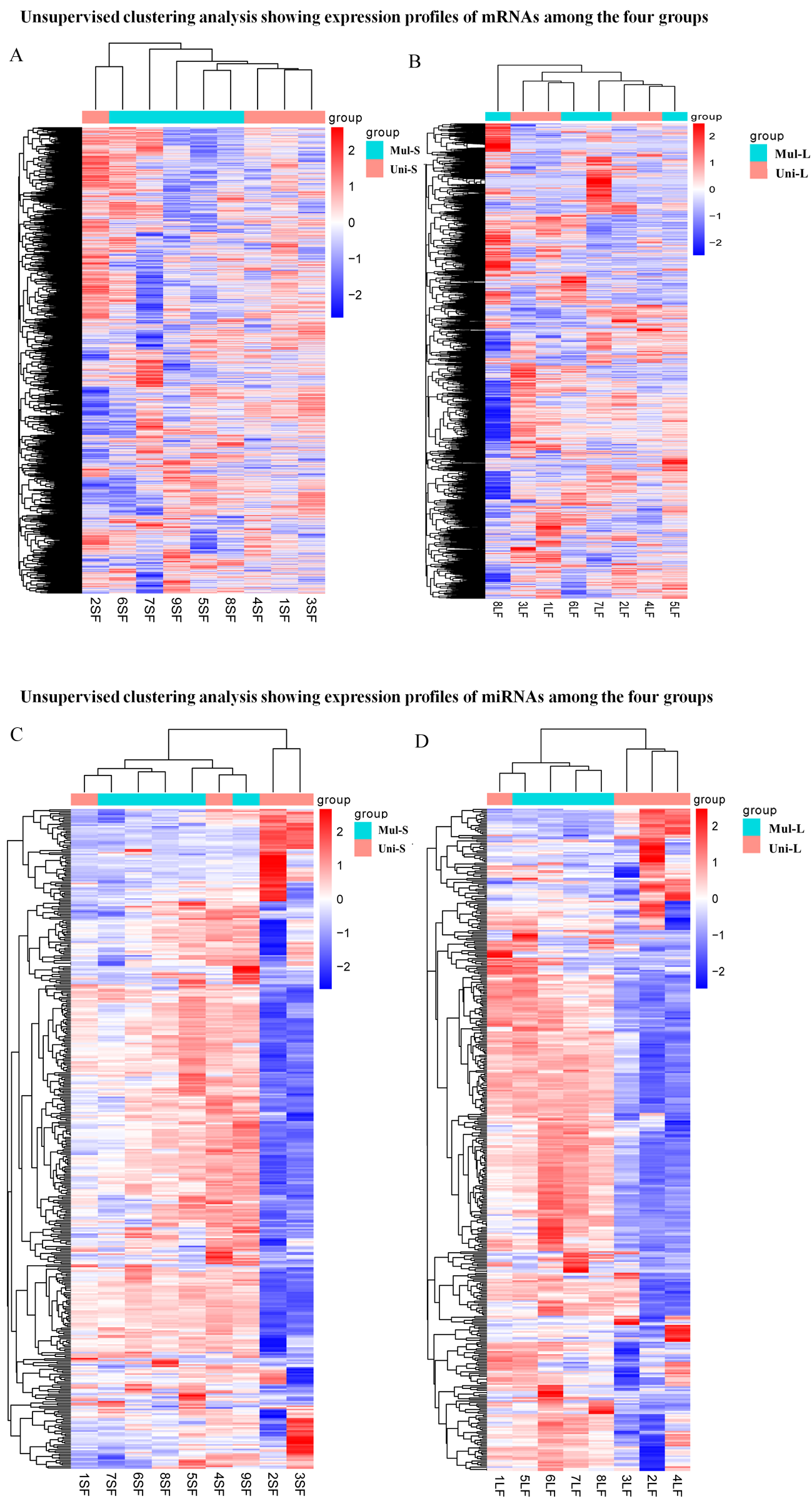

Supplement: Supplementary file 1 — Additional file 1 Figure S1, RNA-seq reveals distinct expression pattern of mRNAs and miRNAs among the four groups. (A) Unsupervised clustering analysis showing expression profiles of mRNAs between Uni-S and Mul-S groups. (B) Unsupervised clustering analysis showing expression profiles of mRNAs between Uni-L and Mul-L groups. (C) Unsupervised clustering analysis showing expression profiles of miRNAs between Uni-S and Mul-S groups. (D) Unsupervised clustering analysis showing expression profiles of miRNAs between Uni-L and Mul-L groups. [file 12864_2020_6671_MOESM1_ESM.tif]
